# Imagery Rescripting: The Impact of Conceptual and Perceptual Changes on Aversive Autobiographical Memories

**DOI:** 10.1371/journal.pone.0160235

**Published:** 2016-08-03

**Authors:** Christien Slofstra, Maaike H. Nauta, Emily A. Holmes, Claudi L. H. Bockting

**Affiliations:** 1 Department of Clinical Psychology and Experimental Psychopathology, University of Groningen, Groningen, The Netherlands; 2 Medical Research Council, Cognition and Brain Sciences Unit, Cambridge, United Kingdom; 3 Department of Clinical Neuroscience, Karolinska Institutet, Stockholm, Sweden; 4 Department of Clinical Psychology, Utrecht University, Utrecht, The Netherlands; University of Tasmania, AUSTRALIA

## Abstract

**Background:**

Imagery rescripting (ImRs) is a process by which aversive autobiographical memories are rendered less unpleasant or emotional. ImRs is thought only to be effective if a change in the meaning-relevant (semantic) content of the mental image is produced, according to a cognitive hypothesis of ImRs. We propose an additional hypothesis: that ImRs can also be effective by the manipulation of perceptual features of the memory, without explicitly targeting meaning-relevant content.

**Methods:**

In two experiments using a within-subjects design (both N = 48, community samples), both Conceptual-ImRs—focusing on changing meaning-relevant content—and Perceptual-ImRs—focusing on changing perceptual features—were compared to Recall-only of aversive autobiographical image-based memories. An active control condition, Recall + Attentional Breathing (Recall+AB) was added in the first experiment. In the second experiment, a Positive-ImRs condition was added—changing the aversive image into a positive image that was unrelated to the aversive autobiographical memory. Effects on the aversive memory’s unpleasantness, vividness and emotionality were investigated.

**Results:**

In Experiment 1, compared to Recall-only, both Conceptual-ImRs and Perceptual-ImRs led to greater decreases in unpleasantness, and Perceptual-ImRs led to greater decreases in emotionality of memories. In Experiment 2, the effects on unpleasantness were not replicated, and both Conceptual-ImRs and Perceptual-ImRs led to greater decreases in emotionality, compared to Recall-only, as did Positive-ImRs. There were no effects on vividness, and the ImRs conditions did not differ significantly from Recall+AB.

**Conclusions:**

Results suggest that, in addition to traditional forms of ImRs, targeting the meaning-relevant content of an image during ImRs, relatively simple techniques focusing on perceptual aspects or positive imagery might also yield benefits. Findings require replication and extension to clinical samples.

## Introduction

The techniques used in psychological therapies typically focus on verbal thought and language. However, mental imagery is an interesting focus for two reasons. First, emotional mental imagery plays an important role in a wide range of psychiatric disorders, including anxiety disorders [1] and depression [2]. Second, experimental studies have demonstrated that imagery elicits a stronger emotional response than verbal thought does [3,4], functioning as an ‘emotional amplifier’ [5] for both positive and negative emotions. Therapeutic techniques that do target mental imagery include imagery rescripting (ImRs; [6]) and Eye-Movement Desensitization and Reprocessing (EMDR; [7]).

Imagery rescripting is used within several psychological therapies including cognitive behaviour therapy and schema focused therapy. The mental image that is being “rescripted” can be an *aversive memory* (the subject of this paper) whereby the problematic image-based memory is transformed into a more benign image [8]. Examples of ImRs for aversive memories include: targeting nightmares and intrusive memories of a trauma event in the treatment of post-traumatic stress disorder (PTSD; [9–11]); intrusive memories in depression [12,13]; and feeling of contamination after childhood sexual abuse [14]. In a review including over 900 participants with various mental health problems, Arntz [6] concludes that the effects of ImRs are promising.

Knowledge about the underlying mechanisms of ImRs is needed [15] and is currently the subject of speculation. The so-called “cognitive” hypothesis of ImRs is that it affects psychopathology by changing “key cognitions” [16], “core beliefs” [17,18] and “negative meanings” [6,19] about the content of the memory. Imagery is also thought to offer an experiential technique to reduce avoidance towards material that has previously been avoided [16]. Thus, by providing access to maladaptive beliefs associated with the image, such beliefs can be modified [20]. Indeed, Long et al. [21] found that ImRs for posttraumatic nightmares led to a decrease in negative trauma-related cognitions and a corresponding decrease in PTSD symptoms, albeit in a small sample. In this literature the term ‘cognition’ refers to semantics—negative meanings associated with the aversive memory. Making changes to this meaning-relevant content of the memory will in this paper be referred to as ‘conceptual changes’ and ‘Conceptual-ImRs’.

While the cognitive hypothesis of ImRs suggests that rescripting “must be closely related to the key cognitions of the patient”([16] p. 445) to be effective, evidence for the necessity of rescripting to be meaning-relevant is lacking. In an experimental trauma-film study in 83 non-clinical volunteers [22], participants were assigned to either a meaning-relevant ImRs condition, a positive imagery condition, or a control condition in which a scene from the traumatic film was recalled and re-experienced. In the meaning-relevant ImRs condition, participants used ImRs to change the script any way they liked and there was a clear connection in meaning for the film memory. In the positive imagery condition a pleasant autobiographical memory that was unconnected to the traumatic film was recalled. Intrusions were only reduced in the active conditions. However, dysfunctional trauma-related cognitions and distress were also more reduced in the positive imagery condition than in the control condition, even though the meaning-relevant content of the film memory was not specifically targeted.

Further doubt upon the necessity of explicitly targeting the meaning-relevant content of an aversive image comes from research into the underlying mechanisms of EMDR, a therapy recognized as an effective treatment for PTSD [23]. In EMDR, patients recall a traumatic memory whilst making horizontal eye-movements. Making eye-movements does not target the (semantic) meaning-relevant content of the memory per se, but it does reduce the unpleasantness and vividness of memories during recall [24]. Also, making eye-movements during recall influences how the memory is stored in long-term memory, rendering subsequent recall less vivid and unpleasant as well ([25]; and for a review of 16 experiments, see [26]). A meta-analysis of 14 clinical studies [27] concluded that the eye-movements component significantly adds to the effectiveness of EMDR. Thus, a technique that does not explicitly target the meaning-relevant content may also significantly contribute to the treatment effect. We note that eye-movements are clearly not the only component of EMDR, and other aspects may also contribute to memory effects with this procedure.

As an alternative to the aforementioned cognitive hypothesis of ImRs, the notion of mental imagery acting as ‘emotional amplifier’ [5] opens up the possibility that other underlying mechanisms of ImRs may be present; namely those capitalizing on imagery properties. The impact of ImRs in changing emotional memories may be explained (at least in part) by directly changing perceptual aspects of the memory representation, and thereby producing a corresponding change in related emotions. Making perceptual changes to an image can involve for example changing colors in the image, or positions of objects. An image can also be changed into an alternative image, by replacing the aversive image with actively generated generic positive imagery (without first making an explicit semantic link to the original image). Neither perceptual changes nor generic positive imagery have to explicitly focus on the original aversive image’s associated cognitions, beliefs, or meanings (meaning-relevant content). Possible advantages of these latter two types of ImRs over traditional ‘conceptual’ ImRs (in which meaning-relevant content of the aversive memory is directly targeted) is that they may be more simple to complete and may possibly require less therapist skill. This may be particularly relevant for patients who appear unable to make healthy changes to the memory themselves [28]. These types of rescripting may also be less burdening to patients, since less focus is placed on the content of the aversive memory itself.

The aim of the current study was to test whether compared to Recall-only and Conceptual-ImRs, a simple form of ImRs, namely Perceptual-ImRs, can reduce the negative aspects of an aversive autobiographical memory such as by merely changing the memory’s perceptual features, without explicitly targeting the meaning-relevant content. In two experiments, we investigated the effects of various ImRs tasks on subsequent recall of aversive autobiographical image-based memories in non-clinical participants. As in laboratory studies of EMDR (e.g. [29]), the effects on unpleasantness and vividness of aversive memories were assessed. Emotionality, the degree to which recall was associated with negative emotions (anxiety, sadness, and helplessness) was also assessed.

In the first experiment, two active manipulations (Conceptual-ImRs and Perceptual-ImRs) were compared to two control conditions, namely a condition in which no ImRs exercise was performed during memory recall (Recall-only; to control for exposure effects) and an active non-visual control condition (Recall+AB; to control for non-specific effects) in which the attention is focused on the breathing (Attentional Breathing). Conceptual-ImRs, that focused on meaning-relevant content of the memory, was operationalized as imagining help and support in the memory. Perceptual-ImRs, that focused on perceptual features of the memory, was operationalized as changing colors, and positions of objects in the memory. It was hypothesized that both ImRs conditions would result in greater decreases in unpleasantness, vividness, and emotionality than Recall-only.

## Materials and Methods Experiment 1

### Participants

Forty-eight students of the University of Groningen (28 female) participated and were remunerated for their time. Participants were recruited via advertisements on social media. The mean age was 23.9 (SD = 2.7). All students over the age of 18 could partake, provided they had not taken part in mindfulness therapy or imagery rescripting in the past. No participants were excluded. One participant dropped out during the training phase. An extra participant was included to complete the data-collection. Ethical approval was obtained from the University of Groningen Ethical Committee of the Psychology Department (ppo-011-247). Participants provided their written informed consent. No data on ethnicity or socioeconomic status were collected. Typically, these samples include a majority of Caucasian students.

### Measures

#### Outcome measures

The outcome measures unpleasantness, vividness, and emotionality of the memory were assessed on 100 mm visual analogue scales (VAS). Participants rated “how unpleasant did you find the memory” on a VAS anchored at each end with “not at all unpleasant” to “extremely unpleasant” (e.g. [30]).

Participants rated “how vivid did you find the memory” on a VAS anchored at each end with “not at all clear” to “extremely clear” (e.g. [30]).

Participants rated to what degree recall of their memory was associated with each of three emotions; anxiety, sadness, and helplessness (from “not at all”, to “extremely”). A mean emotionality score was made from these three ratings.

#### Task compliance measures

Participants rated on 100 mm VAS to what degree they performed each of the manipulation tasks (from “not at all” to “all the time”), with respect to changing colors and positions (relevant to Perceptual-ImRs), imagining help and support (relevant to Conceptual-ImRs), and focusing attention on their breathing (relevant to Recall+AB), and did each of the three ratings for all four conditions.

### Selection of aversive autobiographical memories

For the selection of four (one for each condition) aversive autobiographical memories, a protocol used in previous experiments (i.e. [31]) was followed. Participants were instructed to select several events that made them feel fearful or sad at the time and that still had an emotional impact on them during recall, such as “a farewell”, “being unprepared for an exam” or “witnessing an accident”. They were asked to form a clear specific image of each memory and to briefly describe the image. Participants created labels for these four memories by writing keywords on pieces of paper and then ranked these memories for current emotional impact (from least to most emotional). Based on this ranking, memories were assigned to within-subject conditions, so that the current emotional impact of memories was counterbalanced over conditions.

### Manipulation instructions

The instruction in the Recall-only condition was to “keep the memory in mind”.

The Perceptual-ImRs manipulation consisted of two instructions, namely to “keep the memory in mind and change the colors in the memory” and to “keep the memory in mind and change the positions of objects in the memory”. These instructions were alternated, so that during the manipulation each instruction was presented twice.

The Conceptual-ImRs manipulation consisted of two instructions, namely to “keep the memory in mind and think of what you could say to yourself that would help or support you in the memory and say that to yourself in the memory” and to “keep the memory in mind and bring a helping or supporting other into the memory, think of what this person could do or say to help or support you and let this person do or say that in your memory”. These instructions were alternated, so that during the manipulation each instruction was presented twice.

The instruction in the Recall+AB condition was to “keep the memory in mind and focus on your breathing”.

### Task training

Before the start of the experiment, participants were familiarized with the experiments’ tasks (as in previous research, e.g. [31]). Participants could choose whether they preferred to keep their eyes open or closed during the training, and were instructed to consequently do so throughout the experiment. Participants practiced Perceptual-ImRs (changing colors and positions of objects in a memory) and Conceptual-ImRs (imagining help and support in a memory) on their memory of dinner the day before. Each manipulation task was practiced for 24 s. Participants practiced focusing their attention on their breathing (AB) using a 10 min adaptation from the Dutch translation of the breathing exercise [32]. After the training of each task, participants were asked to rate their training success on a 6-point Likert-scale (0 = not at all, 1 = a little, 2 = somewhat, 3 = reasonably, 4 = good, 5 = very good). If participants rated their performance with “not at all” or “a little”, they were asked to repeat the training and re-rate their performance accordingly.

### Procedure

After the participants were given task training and had selected four aversive autobiographical memories, the experimental phase began. All participants completed all four conditions (Perceptual-ImRs, Conceptual-ImRs, Recall-only, & Recall+AB) of the experiment. The order of conditions was counterbalanced.

In each condition, assessment of the outcome measures (unpleasantness, vividness, and emotionality) took place before and after the experimental manipulation. For this pre- and a post-measurement of the memory, participants were asked to “form an image of the memory and recall this memory as completely and vividly as possible and please indicate when your memory is clear”. After participants said their memory was clear, they were asked to hold the memory in mind for 10 s and then fill out the outcome ratings.

The experimental manipulation (in between the pre- and post-measurements) consisted of four sets of 24 s recall of the aversive memory assigned to that condition, separated by 10 s pause during which participants were instructed to “concentrate on something else”. Participants rated task compliance measures of every manipulation task after each condition.

Participants were free to choose whether they preferred to close their eyes during the AB training and recall of memories or to keep their eyes open, but were instructed to remain consistent in doing so throughout the experiment.

### Design and analyses

A 2 (Time; pre- vs post-measurement) x 4 (Condition; Recall-only vs Perceptual-ImRs vs Conceptual-ImRs vs AB) within-subject design was used. The outcome measures were analyzed with 2 (Time; pre- vs post-measurement) x 4 (Condition; Recall-only vs Perceptual-ImRs vs Conceptual-ImRs vs Recall+AB) repeated-measures ANOVAs. All analyses were two-tailed.

## Results Experiment 1

### Effects of conditions on unpleasantness, vividness, and emotionality

**Fig 1:**
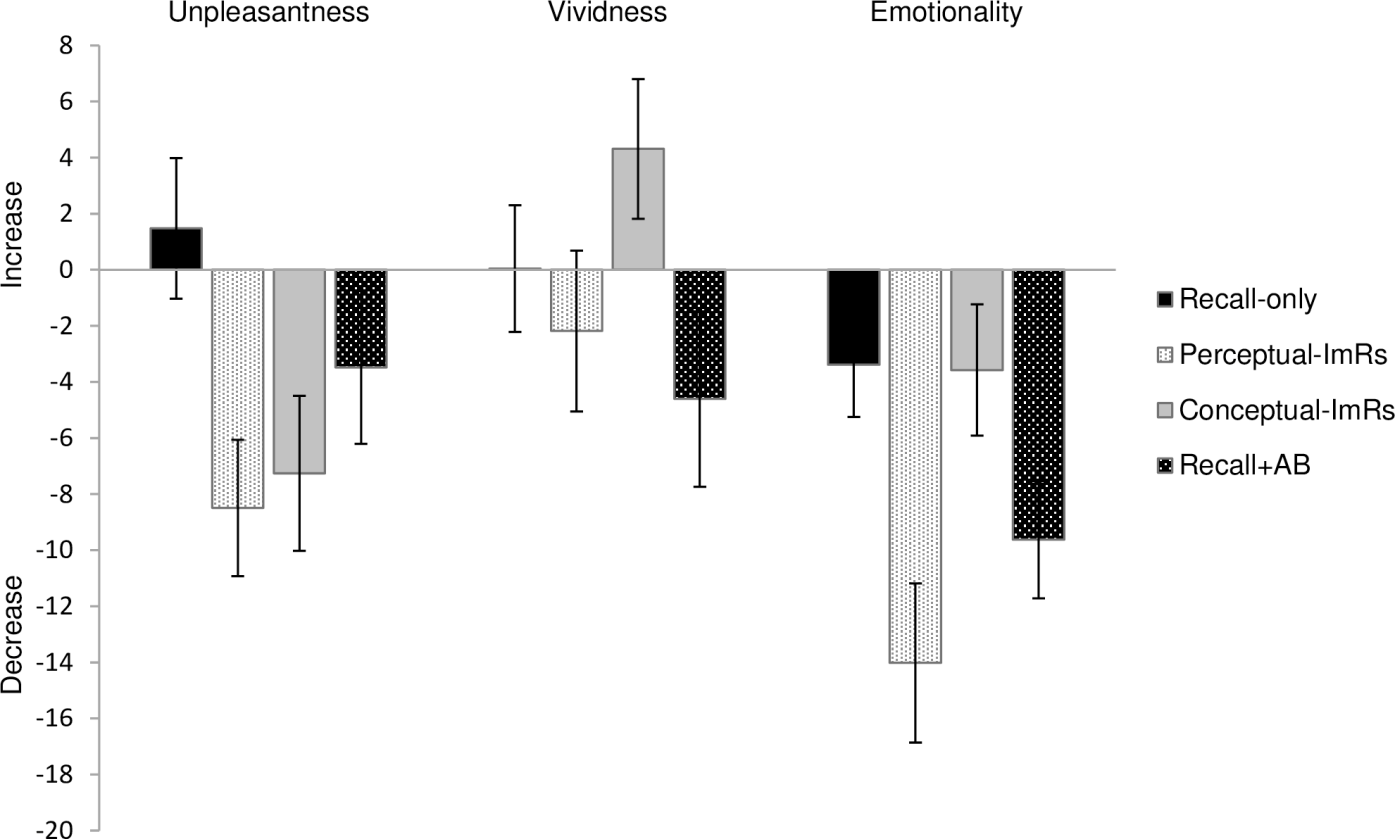
**Changes in unpleasantness, vividness, and emotionality scores for the aversive autobiographical memory per condition** (in mm; post-measurement–pre-measurement; positive scores indicate increases). Bars represent SEM. ImRs = Imagery rescripting, AB = Attentional Breathing.

The difference scores of unpleasantness, vividness, and emotionality are depicted in Fig 1, pre- and post-scores can be found in Table 1. Except for one unusually large decrease in emotionality in the Perceptual-ImRs condition (*z* = -4.14; removing this outlier from the analyses did not significantly impact the results), there were no outliers (−3.29 < *z* < 3.29) in pre-, post-, or difference scores.

**Table 1:** Means (SD) of unpleasantness, vividness, and emotionality of the aversive autobiographical memory at pre- and post-measurement (in mm) per condition.

	Unpleasantness		Vividness		Emotionality	
Condition	Pre	Post	Pre	Post	Pre	Post
Recall-only	53 (24)	54 (24)	66 (22)	66 (22)	54 (26)	47 (27)
Perceptual-ImRs	55 (25)	47 (27)	72 (19)	70 (18)	50 (24)	36 (26)
Conceptual-ImRs	57 (22)	50 (24)	68 (20)	72 (16)	46 (25)	42 (26)
Recall+AB	56 (22)	53 (24)	69 (23)	64 (20)	54 (24)	44 (26)

*Note*. ImRs = Imagery rescripting, AB = Attentional Breathing.

For unpleasantness, there was no significant main effect of Condition (*F*(3,141) = 0.38, *p* = .77, *η*_p_^2^ = .01). The main effect of Time (*F*(1,47) = 10.14, *p* < .01, *η*_p_^2^ = .18) was significant, showing that overall, unpleasantness of memories decreased. Critically, the Condition x Time interaction (*F*(3,141) = 3.10, *p* = .03, *η*_p_^2^ = .06) was significant. Paired *t*-tests revealed that relative to the Recall-only condition, the reductions in unpleasantness were significantly greater in both the Perceptual-ImRs (*t*(47) = 2.81, *p* < .01, *d* = 0.41) and Conceptual-ImRs (*t*(47) = 2.18, *p* = .03, *d* = 0.31) conditions. There was no significant difference in unpleasantness reduction between the Perceptual-ImRs and the Conceptual-ImRs conditions (*t*(47) = 0.38, *p* = .71, *d* = 0.05). Reductions in unpleasantness in the Recall+AB condition did not differ significantly from those in the Recall-only (*t*(47) = 1.32, *p* = .19, *d* = 0.19), nor Perceptual-ImRs (*t*(47) = 1.43, *p* = .16, *d* = 0.21), nor Conceptual-ImRs (*t*(47) = 1.09, *p* = .28, *d* = 0.16) conditions.

For vividness, there was no significant main effect of Condition (*F*(3,141) = 1.04, *p* = .38, *η*_p_^2^ = .02) nor Time (*F*(1,47) = 0.16, *p* = .69, *η*_p_^2^ < .01). The Condition x Time interaction was not significant (*F*(3,141) = 2.17, *p* = .10, *η*_p_^2^ = .04).

For emotionality, there was no significant main effect of Condition (*F*(3,141) = 2.21, *p* = .09, *η*_p_^2^ = .04). The effect of Time (*F*(1,47) = 28.69, *p* < .01, *η*_p_^2^ = .38) was significant, showing an overall decrease of emotionality scores. The critical Condition x Time interaction (*F*(3,141) = 6.01, *p* < .01, *η*_p_^2^ = .11) was also significant. Compared to Recall-only, greater reductions in emotionality were found in the Perceptual-ImRs condition (*t*(47) = 3.10, *p* < .01, *d* = 0.45), but not in the Conceptual-ImRs condition (*t*(47) = 0.07, *p* = .95, *d* = 0.01). The change in emotionality scores in the Perceptual-ImRs condition was significantly larger than the change in the Conceptual-ImRs condition (*t*(47) = 3.29, *p* < .01, *d* = 0.47). The decrease in emotionality in the Perceptual-ImRs condition was not significantly greater than in the active control condition Recall+AB (*t*(47) = 1.34, *p* = .19, *d* = 0.19). The decrease in the Recall+AB condition, in turn, was greater than in the Recall-only (*t*(47) = 2.51, *p* < .01, *d* = 0.36) and Conceptual-ImRs (*t*(47) = 2.33, *p* = .02, *d* = 0.34) conditions.

Whether participants kept their eyes open (N = 18) or closed (N = 30) did not significantly moderate effects of Time or the interaction between Condition and Time for unpleasantness, vividness, or emotionality (all *p*s > .05).

### Task compliance

Table 2 shows task compliance scores per manipulation for each condition. Task compliance was analyzed with separate ANOVAs for the three manipulation tasks. As expected, for the Perceptual-ImRs task (*F*(3,141) = 255.18, *p* < .001, *η*_p_^2^ = .84), Conceptual-ImRs task (*F*(3,141) = 124.89, *p* < .001, *η*_p_^2^ = .73), and AB task (*F*(3,141) = 64.56, *p* < .001, *η*_p_^2^ = .58), the differences between conditions were significant with highest task compliance ratings for the condition-relevant measure. Ratings of condition-relevant tasks were compared and there were no significant differences (*F*(2,94) = 2.41, *p* = .10, *η*_p_^2^ = .05).

**Table 2:** Means (SD) of task compliance measures per condition (in mm). Condition-relevant task compliance ratings are in bold.

Task	Changing colors/positions	Imagining help/support	Attentional Breathing
Condition	Mean (SD)	Mean (SD)	Mean (SD)
Recall-only	9 (15)	22 (28)	33 (31)
Perceptual-ImRs	**73 (19)**	11 (21)	27 (27)
Conceptual-ImRs	5 (8)	**75 (16)**	29 (28)
Recall+AB	7 (15)	14 (23)	**79 (16)**

*Note*. ImRs = Imagery rescripting. AB = Attentional Breathing.

### Discussion Experiment 1

As hypothesized, both ImRs manipulations—Perceptual-ImRs and Conceptual-ImRs—resulted in significantly greater reductions in unpleasantness of the aversive memory than did Recall-only. For emotionality, Perceptual-ImRs—and not Conceptual-ImRs—showed a greater reduction in scores than Recall-only. For vividness, however, there was no significant difference between conditions. Results suggest that simply making perceptual changes to an aversive autobiographical memory, without explicitly targeting the meaning-relevant content, may be effective in terms of changing unpleasantness and emotionality of the memory.

Compared to the active non-visual control condition (Recall+AB), ImRs manipulations did not result in significant decreases in unpleasantness, vividness, or emotionality. Furthermore, Recall+AB did result in decreases in emotionality, compared to Recall-only. Based on these findings, non-specific factors may not be ruled out in understanding (part of) the effectiveness of Imagery Rescripting. If distraction plays a role here in emotion regulation [33], then it is possible that the active control condition also served as a distractor. Further, the working memory hypothesis holds that performing any working memory taxing task [30] during recall of an aversive memory reduces the resources available for this memory, rendering recall less unpleasant and vivid and modifying subsequent recall. If ImRs also taxes working memory [22], a WM hypothesis may also apply to ImRs. An investigation of the possible working mechanisms of ImRs is beyond the scope of this paper. Future studies may seek to examine the role of specific factors in the effectiveness of Imagery Rescripting versus non-specific factors, such as attentional deployment or WM taxing.

Interestingly and unexpectedly, the reductions in emotionality in the Perceptual-ImRs condition were significantly greater than those in the Conceptual-ImRs condition. These results were unexpected and require replication and further examination. In experimental trauma-film studies manipulating the valence of a secondary task during recall, it was found that processing positively valenced material had a greater impact in terms of detail [34], and emotional distress [35]. Possibly, Perceptual-ImRs was a more positively valenced task than Conceptual-ImRs. We note that some other forms of ImRs currently used in therapeutic practice do not start with an aversive memory, but rather patients use their imagination to create positive (adaptive) images *de novo* [8]. It has been argued that increasing positive imagery [36] and positive emotions may be particularly fruitful in depression [37–39]. However, an effect of a generic positive ImRs condition on aversive autobiographical memories may be subject to dispute. While indicated by previous experimental findings [34,35], it stands in contrast to what is predicted by the current cognitive hypothesis of ImRs, which states that “simply asking the patient to imagine some fantastical outcome that could never have happened will not be helpful unless the imagery transformation challenges the toxic meaning of the original memory” ([16] p. 445).

In the second experiment, using a similar within-subjects design, we attempted to replicate the effects of Perceptual-ImRs and Conceptual-ImRs versus a Recall-only control condition. Critically, a positively valenced ImRs condition (Positive-ImRs) was added that focused on actively generating generic (not conceptually or perceptually related to the meaning-relevant content of the aversive autobiographical memory) positive mental imagery during recall. In this experiment, to minimize differences between conditions in terms of difficulty of the ImRs-tasks, participants prepared the changes for all ImRs-tasks before the start of the experiment using labels with keywords (colors for Perceptual-ImRs vs helping others for Conceptual-ImRs vs a fantasy image for Positive-ImRs). We included vividness based on previous studies, but did not formulate a specific hypothesis because of the null finding on vividness in Experiment 1. It was hypothesized that each of the ImRs conditions (Conceptual, Perceptual, and Positive) would result in greater decreases in unpleasantness and emotionality than Recall-only.

## Materials and Methods Experiment 2

### Participants

Forty-eight students (27 female) participated in exchange for remuneration or course-credits. Participants were recruited via posters and the University’s course-credit system. The mean age was 21.7 (SD = 2.3). All students over the age of 18 could partake; no participants were excluded. Ethical approval was obtained from the University of Groningen Ethical Committee of the Psychology Department (12220-N). Participants provided their written informed consent. No data on ethnicity or socioeconomic status were collected. Typically, these samples include a majority of Caucasian students.

### Measures

#### Outcome measures

Identical to Experiment 1.

#### Task compliance measures

Participants rated on 100 mm VAS (from “not at all” to “all the time”) in every condition to what degree they performed each of the ImRs tasks, changing colors (Perceptual-ImRs), imagining help and support (Conceptual-ImRs), and changing the memory into a fantasy image (Positive-ImRs).

### Selection of aversive autobiographical memories

Identical to Experiment 1.

### Manipulation instructions

To prepare participants for the ImRs tasks, they were instructed to create two labels for each ImRs condition (one for the training phase, and one for the experimental phase).

In the Perceptual-ImRs condition, the instruction for the labels was to “name three colors”. The Perceptual-ImRs manipulation consisted of the instruction to “keep the memory in mind and change the colors in the memory”.

In the Conceptual-ImRs condition, the instruction for the labels was to “name a person who supports you and is there for you. Someone you trust”. The Conceptual-ImRs manipulation consisted of the instruction to “keep the memory in mind and bring the helping or supporting other into the memory. Let this person help or support you in your memory”

In the Positive-ImRs condition, the instruction for the labels was to “name a fantasy image. This is a place where you would like to be very much and where you feel completely happy. This can be, but does not have to be, a real place”. The Positive-ImRs manipulation consisted of the instruction to “keep the memory in mind and change it into your fantasy image. Make the fantasy image as positive as possible”.

The instruction in the Recall-only condition was to “keep the memory in mind”.

### Task training

The task training was very similar to Experiment 1, but in Experiment 2 participants practiced Perceptual-ImRs, Conceptual-ImRs, and Positive-ImRs. Training success was again rated for each task (changing colors for Perceptual-ImRs, having someone else help/support you for Conceptual-ImRs, and changing the memory into a fantasy image for Positive-ImRs using a 6-point Likert-scale (0 = not at all, 1 = a little, 2 = somewhat, 3 = reasonably, 4 = good, 5 = very good)).

### Procedure

The only difference in the procedure was that, in line with earlier studies (e.g. [31]) the manipulation in the experimental phase consisted of three times 24 s recall (as opposed to four times 24 s in Experiment 1).

### Design and analyses

A 2 (Time; pre- vs post-measurement) x 4 (Condition; Recall-only vs Perceptual-ImRs vs Conceptual-ImRs vs Positive-ImRs) within-subjects design was used. The changes in unpleasantness, vividness, and emotionality were analyzed with 2 (Time; pre- vs post-measurement) x 4 (Condition; Recall-only vs Perceptual-ImRs vs Conceptual-ImRs vs Positive-ImRs) repeated-measures ANOVAs. All analyses were two-tailed.

## Results Experiment 2

### Effects of conditions on unpleasantness, vividness, and emotionality

**Fig 2:**
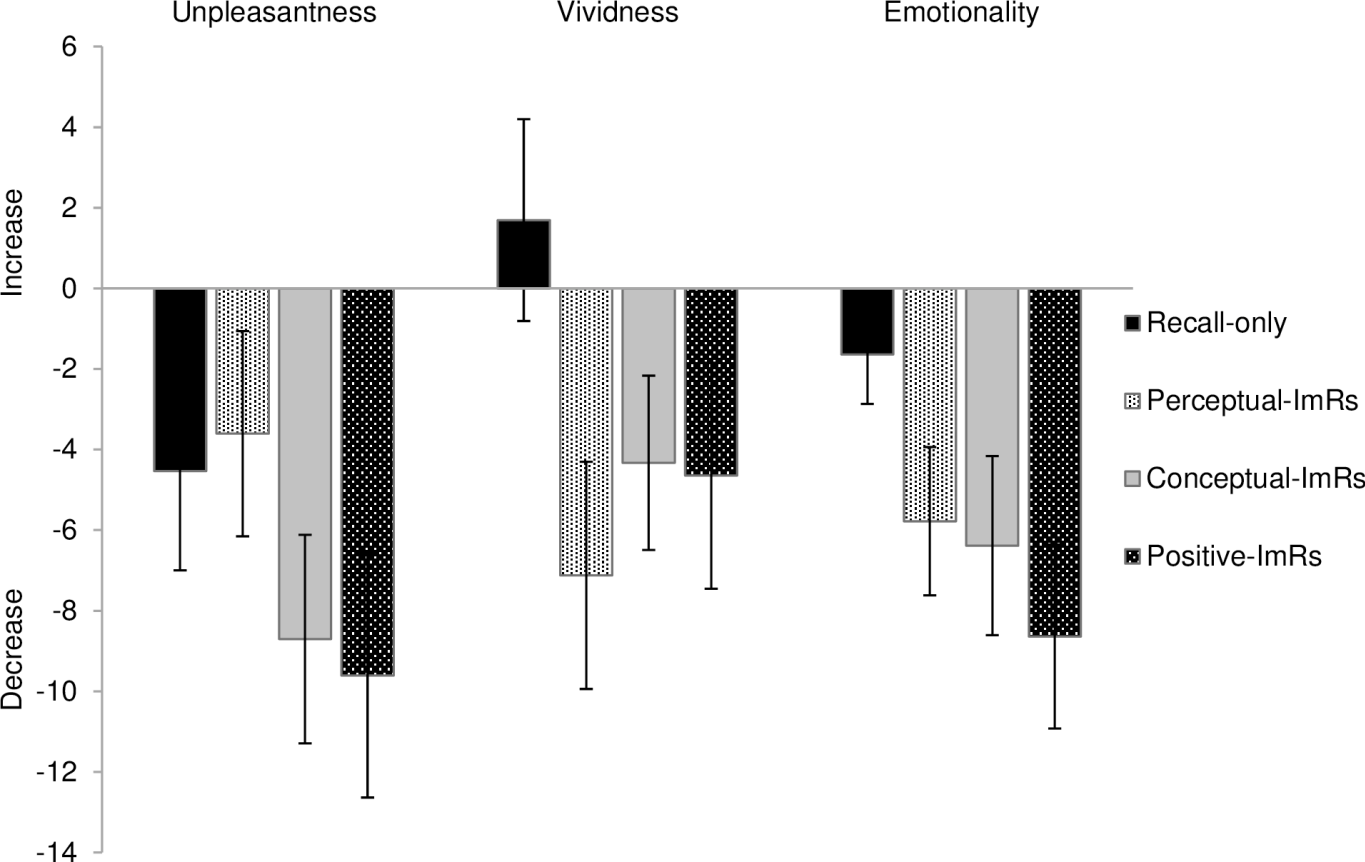
**Changes in unpleasantness, vividness, and emotionality scores per condition** (in mm; post-measurement–pre-measurement; positive scores indicate increases). Bars represent SEM. ImRs = Imagery rescripting.

Pre- and post-measurements of unpleasantness, vividness, and emotionality per condition can be seen in Table 3, and the change scores are depicted in Fig 2. Replacing the two outliers (−3.29 < *z* < 3.29) in pre-scores to deviate 3.29 SD from the mean did not significantly impact the results.

**Table 3:** Means (SD) of unpleasantness, vividness, and emotionality of the memory at pre- and post-measurement (in mm) per condition.

	Unpleasantness		Vividness		Emotionality	
Condition	Pre	Post	Pre	Post	Pre	Post
Recall-only	64 (22)	59 (21)	68 (22)	70 (20)	50 (19)	48 (18)
Perceptual-ImRs	58 (22)	55 (25)	69 (22)	62 (22)	49 (22)	40 (22)
Conceptual-ImRs	63 (21)	54 (21)	76 (13)	72 (15)	51 (21)	45 (21)
Positive-ImRs	65 (19)	56 (23)	74 (16)	69 (19)	50 (19)	41 (22)

*Note*. ImRs = Imagery rescripting.

For unpleasantness, there was no significant main effect of Condition (*F*(3,141) = 0.77, *p* = .51, *η*_p_^2^ = .02). The effect of Time (*F*(1,47) = 16.77, *p* < .001, *η*_p_^2^ = .26) was significant, showing an overall reduction in unpleasantness of memories over time. However, the Condition x Time interaction (*F*(3,141) = 1.48, *p* = .22, *η*_p_^2^ = .03) was not significant.

For vividness, there was an unexpected significant main effect of Condition (*F*(3,141) = 2.84, *p* = .04, *η*_p_^2^ = .06). Paired-samples t-test show that overall vividness scores were lower in the Perceptual-ImRs condition than in the Conceptual-ImRs (*t*(47) = 2.55, *p* = .01, *d* = 0.37) and Positive-ImRs (*t*(47) = 2.14, *p* = .04, *d* = 0.31) conditions. There was no significant difference between conditions at pre-measurement (*F*(3,141) = 2.18, *p* = .09, *η*_p_^2^ = .04). A significant main effect of Time (*F*(1,47) = 8.23, *p* < .01, *η*_p_^2^ = .15) indicates an overall decrease of vividness scores. However, the non-significant Condition x Time interaction (*F*(3,141) = 2.06, *p* = .11, *η*_p_^2^ = .04) demonstrates no difference in decrease of vividness scores between conditions.

For emotionality, there was no significant main effect of Condition (*F*(3,141) = 1.52, *p* = .21, *η*_p_^2^ = .03). The effect of Time (*F*(1,47) = 19.67, *p* < .001, *η*_p_^2^ = .30) was again significant, showing an overall decrease of emotionality scores. The assumption of sphericity for the Condition x Time interaction was violated (*χ*^*2*^(5) = 21.9, *p* < .01). Using the Huynh-Feldt estimates of sphericity (*ɛ* = .83), degrees of freedom were corrected. As predicted, and in line with earlier results, the critical interaction-effect was significant (*F*(2.50,117.45) = 2.97, *p* = .04, *η*_p_^2^ = .06). Compared to Recall-only, emotionality scores were reduced significantly more in the Perceptual-ImRs (*t*(47) = 2.46, *p* = .02, *d* = 0.36), Conceptual-ImRs (*t*(47) = 2.12, *p* = .04, *d* = 0.31), and the Positive-ImRs (*t*(47) = 2.99, *p* < .01, *d* = 0.43) conditions. The reductions in the three ImRs conditions did not differ significantly from each other (ts(47) < 1). Specifically, we tested the difference between Conceptual-ImRs and Perceptual-ImRs (given our findings in Experiment 1), and results showed no significant difference (*t*(47) = 0.23, *p* = .81, *d* = 0.03).

Finally, whether participants kept their eyes open (N = 33) or closed (N = 15) did not interact significantly with effects of Time or the interaction between Condition and Time for any of the outcome measures (all *p*s > .05).

### Task compliance

Task compliance rates (Table 4) show the expected pattern, and were analyzed with separate ANOVAs per task (Recall-only vs Perceptual-ImRs vs Conceptual-ImRs vs Positive-ImRs). Huynh-Feldt correction of degrees of freedom was used because the assumption of sphericity was violated for the Perceptual-ImRs task (*χ*^*2*^(5) = 68.9, *p* < .001; *ɛ* = .61) and the Positive-ImRs task (*χ*^*2*^(5) = 16.6, *p* < .01; *ɛ* = .84). There were significant differences between conditions for the Perceptual-ImRs (*F*(1.83,86.14) = 184.19, *p* < .001, *η*_p_^2^ = .80), Conceptual-ImRs (*F*(3,141) = 130.56, *p* < .001, *η*_p_^2^ = .74), and Positive-ImRs (*F*(2.52,118.37) = 138.80, *p* < .001, *η*_p_^2^ = .75) tasks, with highest compliance ratings for the condition-relevant tasks. The compliance to condition-relevant tasks was compared (correcting degrees of freedom with Huynh-Feldt *ɛ* = .87, as the assumption of sphericity was violated *χ*^*2*^(2) = 9.5, *p* < .01) and there was no significant difference (*F*(1.74,81.80) = 2.97, *p* = .06, *η*_p_^2^ = .06). Thus, all task compliance scores showed the expected manipulation.

**Table 4:** Means (SD) of task compliance measures per condition (in mm). Condition-relevant task ratings are in bold.

Condition	Changing colors	Imagining help/support	Changing into fantastic image
Recall-only	7 (13)	20 (29)	9 (17)
Perceptual-ImRs	**73 (22)**	8 (17)	6 (15)
Conceptual-ImRs	7 (11)	**82 (20)**	12 (23)
Positive-ImRs	14 (24)	16 (25)	**77 (25)**

*Note*. ImRs = Imagery rescripting.

## Discussion Experiment 2

As hypothesized, Conceptual-ImRs, Perceptual-ImRs, and Positive-ImRs all resulted in greater decreases in emotionality than Recall-only. For vividness, there was an overall decrease in vividness scores over time. However, reductions in vividness were not significantly different between conditions; the crucial interaction effect was not significant, as in Experiment 1. Vividness scores in the Perceptual-ImRs condition were lower overall, which may have reduced possible change in this condition (floor effect). At pre-measurement, however, there was no significant difference between conditions. Possibly, including three active conditions in the analyses may have obscured small differential effects on vividness.

The effects on unpleasantness and the unexpected finding of superiority of Perceptual-ImRs over Conceptual-ImRs on emotionality in Experiment 1 were not replicated in Experiment 2. Experiment 1 and 2 had highly similar designs and procedures, which renders it unlikely that the minor changes in the procedure explain discrepancies in results. These disparities in results do not necessarily reflect significant differences between the experiments and may merely represent chance findings. The finding that was consistent across both studies, reduction of emotionality to Perceptual-ImRs, appears more robust. Nevertheless is it important to acknowledge those findings that did not replicate. Future studies may seek to replicate the intriguing superiority of specific ImRs tasks.

## General Discussion

A cognitive hypothesis of ImRs suggests that it is necessary to target the meaning-relevant content of an image for ImRs to be successful [16]. This seems at odds with the findings in these studies that Perceptual-ImRs and Positive-ImRs also resulted in beneficial effects. In Experiment 1, compared to Recall-only, Conceptual-ImRs and Perceptual-ImRs both resulted in greater decreases in memory’s unpleasantness. Further, Perceptual-ImRs—but not Conceptual-ImRs—resulted in greater decreases in memory-associated emotionality than Recall-only. In Experiment 2, Conceptual-ImRs, Perceptual-ImRs, and Positive-ImRs resulted in greater decreases in memory-associated emotionality than Recall-only. This is consistent with our alternative hypothesis that simply focusing on perceptual features of an aversive memory may also affect the memory and associated emotions.

Reductions in unpleasantness and emotionality did not co-occur with significant effects on vividness in either Experiment 1 or 2. This corresponds to the findings of Tadmor et al. [35], that a positively valenced concurrent task affected emotional distress, but not vividness, using a trauma-film paradigm. Tadmor et al. [35] propose that evoking positive emotions during recall may specifically counteract the unpleasant memory’s negative emotionality, without affecting its vividness. Both Perceptual-ImRs and Positive-ImRs seem to be creative, entertaining, and inherently positive tasks that bring about positive emotions. Interestingly, these positive emotions may defy the meaning-relevant content of the image indirectly, for example by providing corrective valence-incongruent information [40]. Also, such a positive experience of recall may defy meta-cognitive appraisals patients may have about their images, such as “having this image means I’m going crazy” or “having this image means I will lose control”. Thus, the beneficial effects of both Perceptual-ImRs and Positive-ImRs may still be accounted for by a (albeit modified or meta-) cognitive hypothesis of ImRs. Perceptual-ImRs and Positive-ImRs may offer a positive experience of recall that defy the original aversive image’s associated cognitions, beliefs, or meanings (meaning-relevant content), although they do not verbally discuss them directly. Future studies should seek to test this.

The null-finding on vividness stands in contrast, however, to previous findings with concurrent tasks during recall. Usually these concurrent tasks directly affect vividness, but not unpleasantness [25]. Also, a time-course study suggests that concurrent tasks first impact vividness, which in turn renders the emotional memory less unpleasant [41]. There may be mechanisms at work differentially targeting vividness and emotionality while performing concurrent tasks during recall. Based on the present data this matter remains speculative. It has been observed that it may be a matter of power that effects of performing a concurrent task during recall often only manifest on either unpleasantness or vividness [31].

### Limitations and future directions

The current experiments investigated the effects on aversive memories in a non-clinical sample of young adults. Participants in the current study were free to choose anxious or sad memories, as in previous studies investigating the effects of therapeutic techniques on aversive memories (e.g. [31]). The aversive autobiographical memories chosen by participants, such as the death of a grandparent, an argument with a friend, or witnessing an accident, were not necessarily intrusive (i.e. did not come to mind involuntarily) and not extremely unpleasant (mean pre-measurement around 60 mm on a 100 mm VAS). This limits the possible change, and thus the sensitivity of the measure. However, the range of unpleasantness was well within the range of earlier studies in analogue groups [42]. It also limits the generalizability to other types of images in clinical samples. Therefore, replication studies in patient samples are warranted. In these clinical samples, the tolerability and feasibility of different ImRs techniques should be assessed.

The effects of ImRs tasks may be moderated by image characteristics. For example, they may be different for traumatic memories, as some researchers claim that traumatic memories differ qualitatively from normal aversive memories [43], although others also suggest that they are on a continuum [44,45]. Further, it has been suggested that ImRs, compared to imaginal exposure, may be especially suited for targeting non-fear emotions [20,46], although recent experimental studies found no differential effects [47,48]. Additional analyses in this study revealed that the relative (to anxious) sadness of the memory (at pre-measurement) did not moderate the results in Experiment 1 or 2 (all *p*s > .05). This is in line with findings that ImRs may be effective in targeting a wide range of mental images [6]. Exploring potential moderators of effect for different ImRs techniques in future research may inform tailoring of diverse ImRs techniques to individual needs and memory characteristics [6].

Furthermore, the within-subjects design used in this study poses the restriction that results are limited to effects on unpleasantness, vividness, and emotionality of memories, whereas ImRs aims to target more aspects of psychopathology. The question remains how these short-term effects on laboratory measures translate to long-term effects on images and psychopathology in patients’ daily life. A between-subjects design would allow for follow-up measurements or time series of the imagery and associated emotions and cognitions, as well as targets of ImRs, such as frequency and quality of intrusions, symptom levels of PTSD and depression. This could elucidate the dynamics between these variables over time and give insight into if and how ImRs might attenuate these dynamics.

Finally, generalizability of the effects of the ImRs instructions in this experiment to the therapeutic intervention of ImRs as clinically practiced is limited. The ImRs instructions in this study were concise and restrictive to produce reliable manipulation of the memories, but there are clearly many different ways to alter an image. Having participants describe afterwards how they rescripted their memories would have given an extra manipulation check to ensure that participants did change meaning-relevant content in the Conceptual-ImRs condition and did not do so in the Perceptual-ImRs and Positive-ImRs conditions. In future research, these descriptions could be used for dose-response analyses and also provide insightful additional qualitative information.

In conclusion, this study provides preliminary evidence that ImRs can be effective (at least on unpleasantness and emotionality) without specifically or explicitly targeting meaning-relevant content, but by simply targeting perceptual features or generating positive imagery. If extended to patient samples, this may be a fruitful avenue towards developing more simple and more tolerable ImRs techniques.
